# Circ_0000479 promotes proliferation, invasion, migration and inflammation and inhibits apoptosis of rheumatoid arthritis fibroblast-like synoviocytes via miR-766/FKBP5 axis

**DOI:** 10.1186/s13018-023-03700-0

**Published:** 2023-03-21

**Authors:** Peng Zhao, Guobin Ma, Lintong Ma

**Affiliations:** 1 Department of Rheumatology Immunology, 3201 Hospital, 783 Tianhan Avenue, Hantai District, Hanzhong, 723000 China; 2 Department of Hematology, 3201 Hospital, Hanzhong, China

**Keywords:** circ_0000479, miR-766, FKBP5, Rheumatoid arthritis

## Abstract

Circular RNAs have been demonstrated to play a critical role in the progression of autoimmune diseases. This study aimed to investigate the function of circ_0000479 in rheumatoid arthritis (RA) fibroblast-like synoviocytes (FLSs). Circ_0000479 was found to be upregulated in RA-FLSs. Flow cytometry analysis, cell counting Kit-8, transwell, wound-healing and enzyme-linked immunosorbent assays were conducted to evaluate RA-FLS apoptosis, proliferation, invasion, migration and inflammation. The results confirmed that circ_0000479 knockdown suppressed pathogenic properties of RA-FLSs. Through bioinformatics analysis and screening, we obtained 18 miRNAs that can bind to circ_0000479, of which miR-766 was most significantly up-regulated after circ_0000479 knockdown. MiR-766 was confirmed to be down-regulated in RA-FLSs and the combination between circ_0000479 and miR-766 was verified by dual-luciferase reporter assays. Moreover, the inhibitory effect of circ_0000479 knockdown in RA-FLS progression was attenuated by miR-766 inhibitor. By intersecting the target genes of miR-766 with the up-regulated genes in RA, we obtained 8 genes, of which FKBP5 was most significantly down-regulated after miR-766 overexpression. The results of dual-luciferase reporter assays also verified that FKBP5 was the target gene of miR-766. In addition, FKBP5 overexpression abated the inhibition of RA-FLS progression caused by circ_0000479 silencing. In summary, circ_0000479 binds to miR-766 to promote RA progression via FKBP5.

## Introduction

Rheumatoid arthritis (RA) is a chronic, systemic autoimmune disease with erosive, symmetrical polyarthritis as the main clinical manifestation [1, 2]. The basic pathological changes are chronic inflammation of the joint synovium, the formation of pannus, and the gradual destruction of articular cartilage and bone, eventually leading to joint deformity and loss of function [3]. Substantial evidence suggests that, as key cells involved in the formation of synovial tissue, fbroblast-like synoviocytes (FLSs) plays an important role in maintaining and exacerbating joint inflammation [4, 5]. Recent studies have shown that distinct FLS subsets expand and drive inflammation and damage in RA [6, 7]. Therefore, research on the pathological mechanism of FLSs is crucial for finding novel RA diagnosis and treatment methods.

Circular RNAs (circRNAs) are a class of non-coding RNA molecules with regulatory functions that form a circular structure by covalent bonds [8, 9]. Some circRNAs containing miRNA response elements can act as competitive endogenous RNAs to bind to miRNAs, thereby releasing the inhibitory effect of miRNAs on their target genes and upregulating the expression levels of their target genes [10–12]. CircRNAs play crucial roles in the biological processes of a variety of human diseases, such as autoimmune diseases [13, 14] and cancer [15, 16]. Due to the insensitivity to nucleases, circular RNAs are more stable than linear RNAs, which makes circular RNAs have obvious advantages as novel clinical diagnostic markers.

Recent studies have shown that some circRNAs are involved in occurrence and development of RA [17]. Hsa_circ_0044235 was found to regulate NLRP3-mediated pyroptosis through the miR-135b-5p-SIRT1 axis, thereby regulating the development of RA [18]. Circ_0008360 inhibits FLS proliferation, migration and inflammation and promotes apoptosis by regulating the miR-135b-5p/HDAC4 axis in rheumatoid arthritis, providing a potential target for the prevention and treatment of RA [19].Circ_0000479 was found to be significantly up-regulated in patients with systemic lupus erythematosus (SLE) and may serve as a potential biomarker for its diagnosis [20, 21]. Therefore, we speculate that circ_0000479 may also play a role in the pathogenesis and progression of RA, which is also an autoimmune disease.

In this study, we first detected the expression of circ_0000479 in RA-FLSs and normal FLSs, and verified its stability. We then investigated the effect of circ_0000479 knockdown on the proliferation, invasion, migration, inflammation and apoptosis of RA-FLSs. Subsequently, we screened miRNAs that bind to circ_0000479 and explored the mechanism of circ_0000479 in regulating RA progression through miR-766.

## Materials and methods

### Cell culture and transfection

RA-FLSs (MH7A cells) and normal FLSs (primary cells) were purchased from Wuhan Procell Life Technology Co., Ltd. Cells were cultured in DMEM (Invitrogen) containing 10% FBS (Invitrogen) and 1% penicillin–streptomycin (Invitrogen) at 37 °C with 5% CO_2_. Small interfering RNA of circ_0000479 (si-circ_0000479) and scramble control si-NC, miR-766 mimics (miR-766) and related control miR-NC, miR-766 inhibitor and FKBP5 overexpression vector (FKBP5) were synthesized by GenePharma (Shanghai, China). MH7A cells were plated into 6-well plates and transfected with the indicated compositions using Lipofectamine 2000 (Invitrogen). The initial concentration of siRNA is 20 μM, the initial concentration of miRNA mimic and miRNA inhibitor is 50 μM. When used, it was diluted 1000 times, namely 20 nM and 50 nM respectively.

### Quantitative real‑time polymerase chain reaction (qRT‑PCR)

Total RNA was extracted from cultured cells with TRIzol reagent (Invitrogen) and cDNA synthesis was conducted on the RNA using Reverse Transcription System Kit (Takara, Dalian, China). SYBR Premix Ex Taq (Takara Biotech, Japan) on an ABI 7900 system (Applied Biosystems, Foster City, CA, USA) was employed to conduct the amplification of cDNAs. GAPDH was used as endogenous control, and the relative expression was calculated by a 2^−ΔΔCt^ method. The primers were shown in Table 1.

**Table 1:** List of primers used in this study

Gene		Sequence(5'–3')
Circ_0000479	Forward	AAGAGAAGAATCTGTAAGAATCA
	Reverse	TGGTGCTATCAAGGTGTA
EPSTI1	Forward	ATCTTGAGACTCGCTAAGCGTCCCAG
	Reverse	TCAACAGCACTAACACAGCTTCAG
FKBP5	Forward	TGGCCATGTGCTACCTGAAGCT
	Reverse	CTGAAATGTGCTGGACTTAAGCTG
RSG16	Forward	GCTTGCAGGC TGCTAAACCCAA
	Reverse	CACTTAAATAATCTCCTGG
ELL2	Forward	CGACC TTCAATCCAGTTCCAA
	Reverse	TGCTGGTCCTCATAGATACT
MYO6	Forward	GGATCTG TCCGAGCAGGAAGCC
	Reverse	ATCACCATGGTCATCATGCA
ATP1A2	Forward	TCC GACTGTCCCAGACGGGCTGG
	Reverse	GAGACCG CGTCCCTGCT GACCT
HIF3A	Forward	TGTGGAGTCATCTCACCGCC
	Reverse	TGGCGTTGAGCTGGAAGTCA
NRG2	Forward	GATCCTGTGCACTGACTGCG
	Reverse	AGAACGTTCCAGGCTCTGGACG
GAPDH	Forward	CGGAGTCAACGGATTTGGTC
	Reverse	CGGTGCCATGGAATTTGCCA
U6	Forward	GCTTCGGCAGCACATATACTAA
	Reverse	AACGCTTCACGAATTTGCGT

### RNase R treatment

To test whether circ_0000479 has higher stability than linear RNA molecules, we treated RNA with RNaseR (Epicenter, Madison, WI, USA), a 3'-5' exonuclease that digests almost all linear RNAs, but not circRNAs. 10U RNase R was added to 2.5 μg total RNA extracted from MH7A cells, incubated at 37 °C for 30 min, and then qRT-PCR was performed to quantify the enrichment of circ_0000479 and EPSTI1.

### Actinomycin D treatment

MH7A cells were seeded in six-well plates (5 × 10^5^ cells per well). Twenty-four hours later, cells were exposed to 2 μg/ml Actinomycin D (Sigma) and collected at indicated time points. The RNA levels of circ_0000479 and EPSTI1 were analyzed using qRT-PCR and normalized to the values measured in the 0 h group.

### Subcellular fraction analysis

Nuclear and cytoplasmic fractions in MH7A cells were isolated using PARIS reagent (Ambion, Austin, TX, USA) according to the manufacturer's instructions. RNA isolated from the fractions was then subjected to qRT-PCR to examine the levels of circ_0000479, GAPDH (cytoplasmic control transcript) and U6 (nuclear control transcript).

### Cell counting Kit-8 (CCK-8) assays

MH7A cells were cultured in 96-well plates and separately incubated for 0, 24, 48 and 72 h. In accordance with the manufacturer's protocol, optical density (OD) values were measured using the CCK-8 solution (Beyotime, Shanghai, China). The absorbance values at each point were measured at 450 nm. Each experiment was independently repeated at least three times.

### Flow cytometry analysis

After 24 h incubation, MH7A cells were resuspended in binding buffer and dual-labeled with Annexin V-fuorescein isothiocyanate and propidium iodide reagent (Beyotime) for 20 min without light. The apoptotic rate was determined by the flow cytometer (Beckman Coulter, Brea, CA, USA).

### Transwell assays

Transwell assays were carried out to assess the invasive ability of MH7A cells. Transfected MH7A cells cultured without FBS were inoculated on Matrigel-coated upper chambers and the culture medium containing 20% FBS was added into the lower chambers. After 24 h incubation, the uninvaded cells were removed. The filter was then fixed with 4% paraformaldehyde for 20 min and dyed with crystal violet for 15 min at 25 °C. The cell invasion images were captured by inverted microscope at a magnification of 100 × .

### Scratch wound healing assays

MH7A cells were grown in 6-well plates and cultured with RPMI-1640 supplemented with 10% FBS overnight. We drew three parallel lines on the back of the 6-well plate, and then used a 10 μl pipette tip perpendicular to the parallel line to scratch a wound. The cells were washed with PBS to remove the floating cells and then cultured in fresh medium. After 24 h incubation, the cell migration images were captured by inverted microscope at a magnification of 40 × .

### Western blot analysis

Total proteins were extracted using cell lysis buffer (Beyotime) and thirty micrograms of proteins were subjected to 12% SDS-PAGE electrophoresis. The samples were then transferred to the nitrocellulose membranes and blocked with 5% non-fat milk for 1.5 h. The protein band, specifically bound to the primary antibody, was detected through the FluorChem imaging system (alpha innotec GmbH, Kasendorf, Germany). The primary antibodies were FKBP5 (ab126715, Abcam), EPSTI1 (ab233036, Abcam) and GAPDH (ab9485, Abcam).

### Enzyme‑linked immunosorbent assays (ELISA)

ELISA is a solid-phase immunoassay method for the detection of trace substances. MH7A cells were seeded in 6-well plates and incubated for 24 h. We then measured the concentrations of TNF-α, IL-6 and IL-1β in the supernatants of MH7A cells via TNF-α (ab181421, Abcam), IL-6 (ab178013, Abcam) and IL-1β (ab214025, Abcam) ELISA kits according to the protocols.

### Dual‑luciferase reporter assays

Dual-luciferase reporter assays were carried out using the Dual-luciferase Reporter Assay System (Promega, Madison, WI, USA). Briefly, we constructed pMIR-reporter luciferase vectors containing a specific sequence of wild-type (WT) or mutant (MUT) fragment of circ_0000479 or FKBP5. Next, we co-transfected these vectors and miR-NC/miR-766 into MH7A cells. Cells were then collected and lysed for luciferase detection 48 h after transfection. The relative luciferase activity was normalized against to the Renilla luciferase activity.

### Statistics

We used GraphPad Prism 7 for data analysis. Results were presented as mean ± SD. Each experiment was independently conducted triple times. Analysis of differences was performed via unpaired Student’s t-test or one-way ANOVA. A probability of 0.05 or less was considered statistically significant.

## Results

### Circ_0000479 was upregulated in MH7A cells

**Fig. 1:**
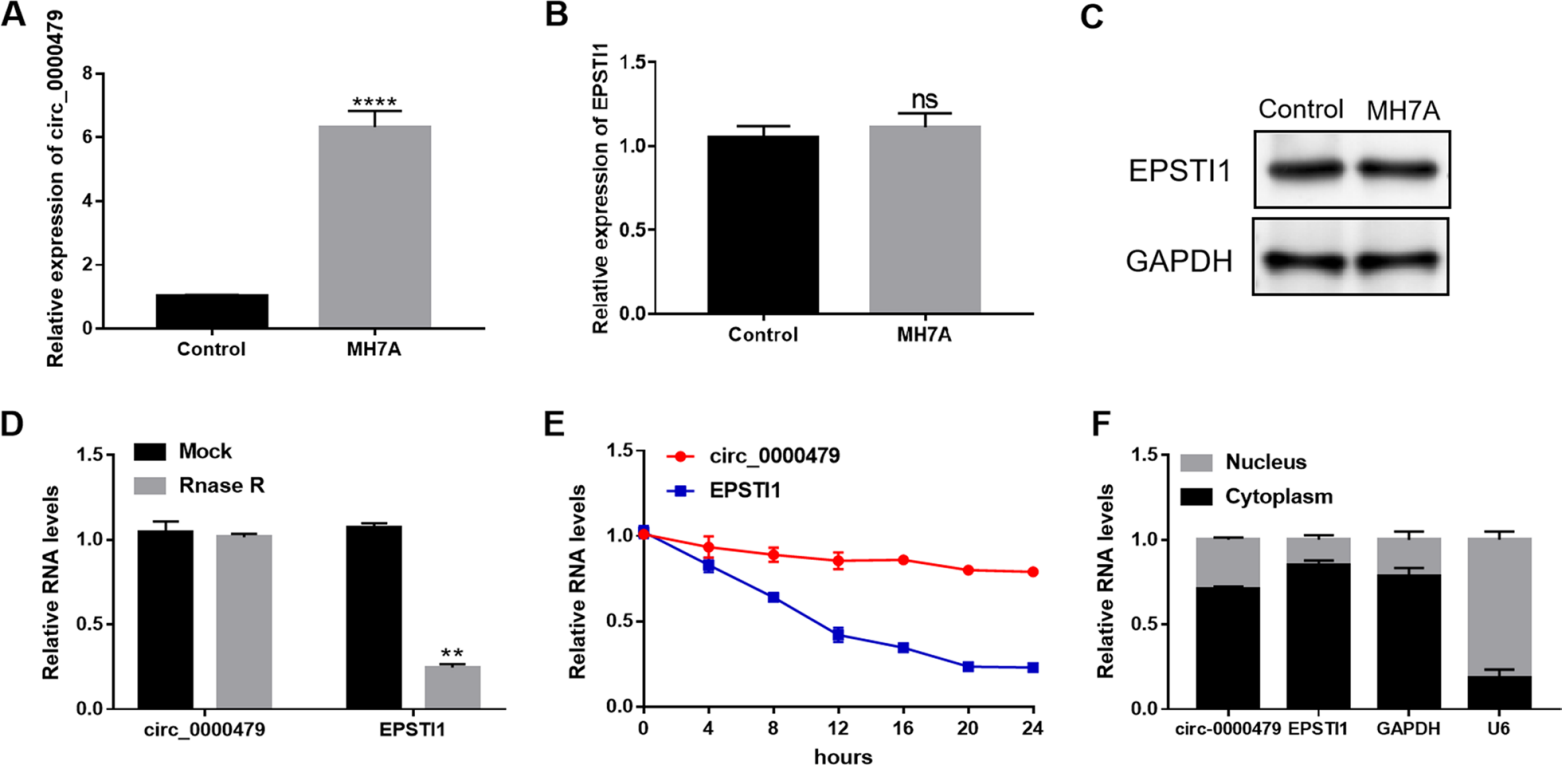
Circ_0000479 was upregulated in MH7A cells. **A** Relative expression of circ_0000479 in MH7A cells and normal FLSs. **B** Relative expression of EPSTI1 in MH7A cells and normal FLSs. **C** The expression of EPSTI1 protein in MH7A cells and normal FLSs. **D** Relative RNA levels of circ_0000479 and EPSTI1 after RNase R treatment. **E** Analysis for RNA abundance of circ_0000479 and EPSTI1 after Actinomycin D treatment at indicated time points. **F** Detected by qRT-PCR, circ_0000479 was mainly enriched in the cytoplasm. **P < 0.01; ****P < 0.0001

To construct an RA model in vitro, we cultured MH7A cells—an immortalized human rheumatoid FLS line derived from the soft tissue of the knee joint of RA patients [22]. We first detected the expression of circ_0000479 in MH7A and normal FLSs. As shown in Fig. 1A, compared to normal FLSs, circ_0000479 was highly expressed in MH7A cells. EPSTI1 is the parental gene of circ_0000479 and EPSTI1 was not differentially expressed at the gene and protein levels between MH7A cells and normal FLSs (Fig. 1B, C). To investigate whether circ_0000479 is more stable than linear EPSTI1, we then conducted RNase R and actinomycin D assays. The results of RNase R assay illustrated that RNase R could not digest circ_0000479, but significantly digested linear EPSTI1 (Fig. 1D). Treatment by Actinomycin D showed that the half-life of circ_0000479 was significantly higher than that of linear EPSTI1 (Fig. 1E). The above two experiments indicated that circ_0000479 has higher stability than its parental gene EPSTI1 and circ_0000479 is a true circRNA. Through subcellular fraction analysis, circ_0000479 was found to be mainly enriched in the cytoplasm (Fig. 1F).

### Circ_0000479 knockdown suppressed MH7A cell progression

**Fig. 2:**
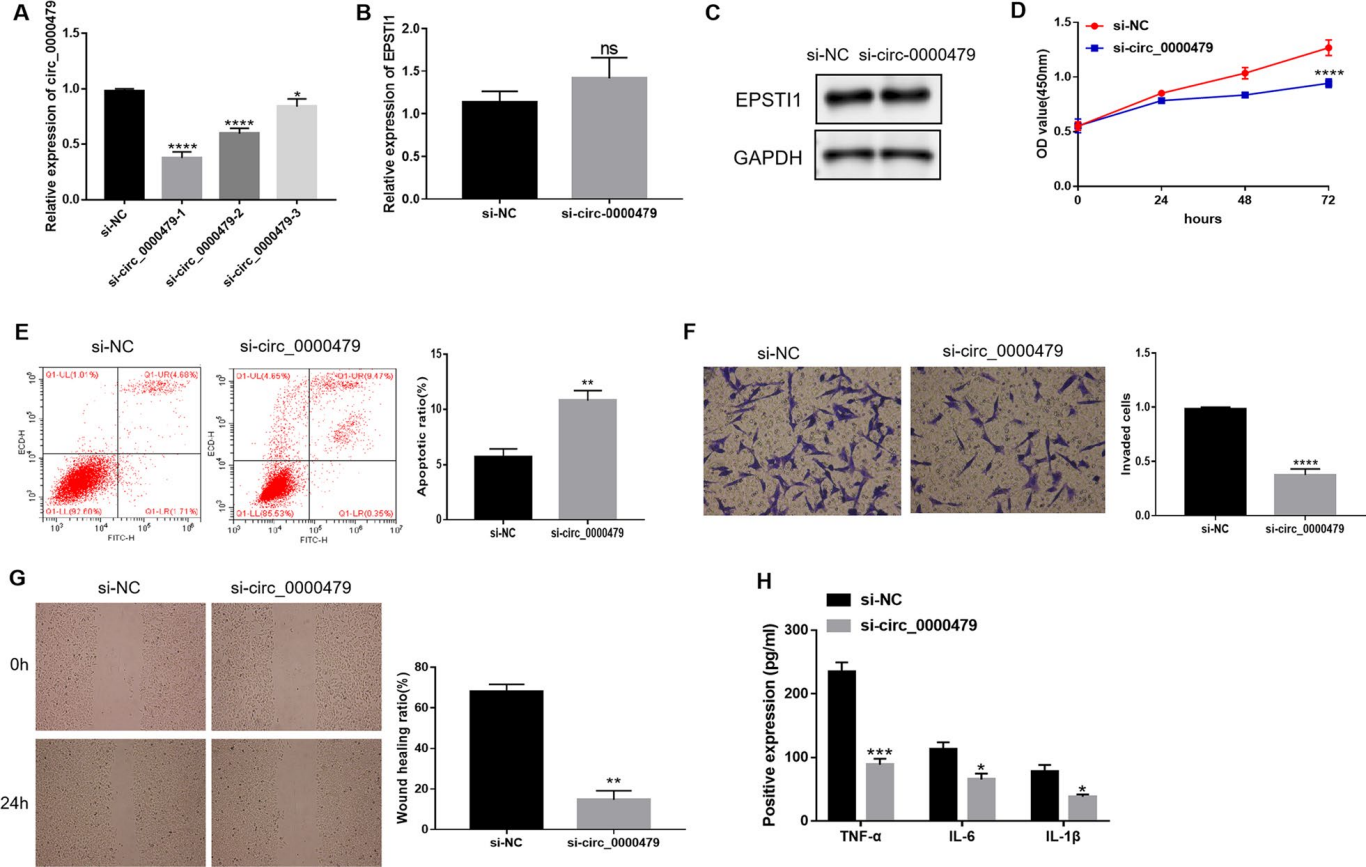
Effects of circ_0000479 on MH7A cell progression. **A** The screening of interfering fragments of circ_0000479 by qRT-PCR. **B** Relative expression of EPSTI1 in MH7A cells transfected with si-circ_0000479 or si-NC. **C** The expression of EPSTI1 protein in MH7A cells transfected with si-circ_0000479 or si-NC. **D** CCK-8 assays detected the OD value of MH7A cells transfected with si-circ_0000479 or si-NC. **E** The apoptotic ratio of MH7A cells transfected with si-circ_0000479 or si-NC by flow cytometry analysis. **F** Transwell assays detected the number of invaded MH7A cells after transfection of si-circ_0000479 or si-NC. Magnification: 100 × . **G** The wound healing ratio of MH7A cells after transfection of si-circ_0000479 or si-NC. Magnification: 40 × . **H** The positive expression of TNF-α, IL-6 and IL-1β in MH7A cells transfected with si-circ_0000479 or si-NC by ELISA kits. *P < 0.05; **P < 0.01; ***P < 0.001; ****P < 0.0001

To investigate the function of circ_0000479 in RA progression, MH7A cells were transfected with si-circ_0000479 to interfere the expression of circ_0000479. As shown in Fig. 2A, identified by qRT-PCR, si-circ_0000479-01 was found to have the best interference effect and was used for subsequent studies. The results of qPCR and Western blot showed that si-circ_0000479 could not affect the expression of EPSTI1 (Fig. 2B, 2C). As demonstrated by the result of CCK-8 assays, the proliferation of MH7A cells was inhibited by silencing circ_0000479 (Fig. 2D). Flow cytometry analysis showed that circ_0000479 knockdown promoted the apoptosis of MH7A cells (Fig. 2E). Compared with the si-NC control group, we found that circ_0000479 deficiency inhibited the invasion and migration of MH7A cells by transwell and wound healing assays, respectively (Fig. 2F, 2G). Furthermore, circ_0000479 knockdown decreased the concentrations of TNF-α, IL-6 and IL-1β in MH7A cells (Fig. 2H). These findings suggested that circ_0000479 knockdown inhibited the progression of MH7A cells.

### Circ_0000479 sponged miR-766

**Fig. 3:**
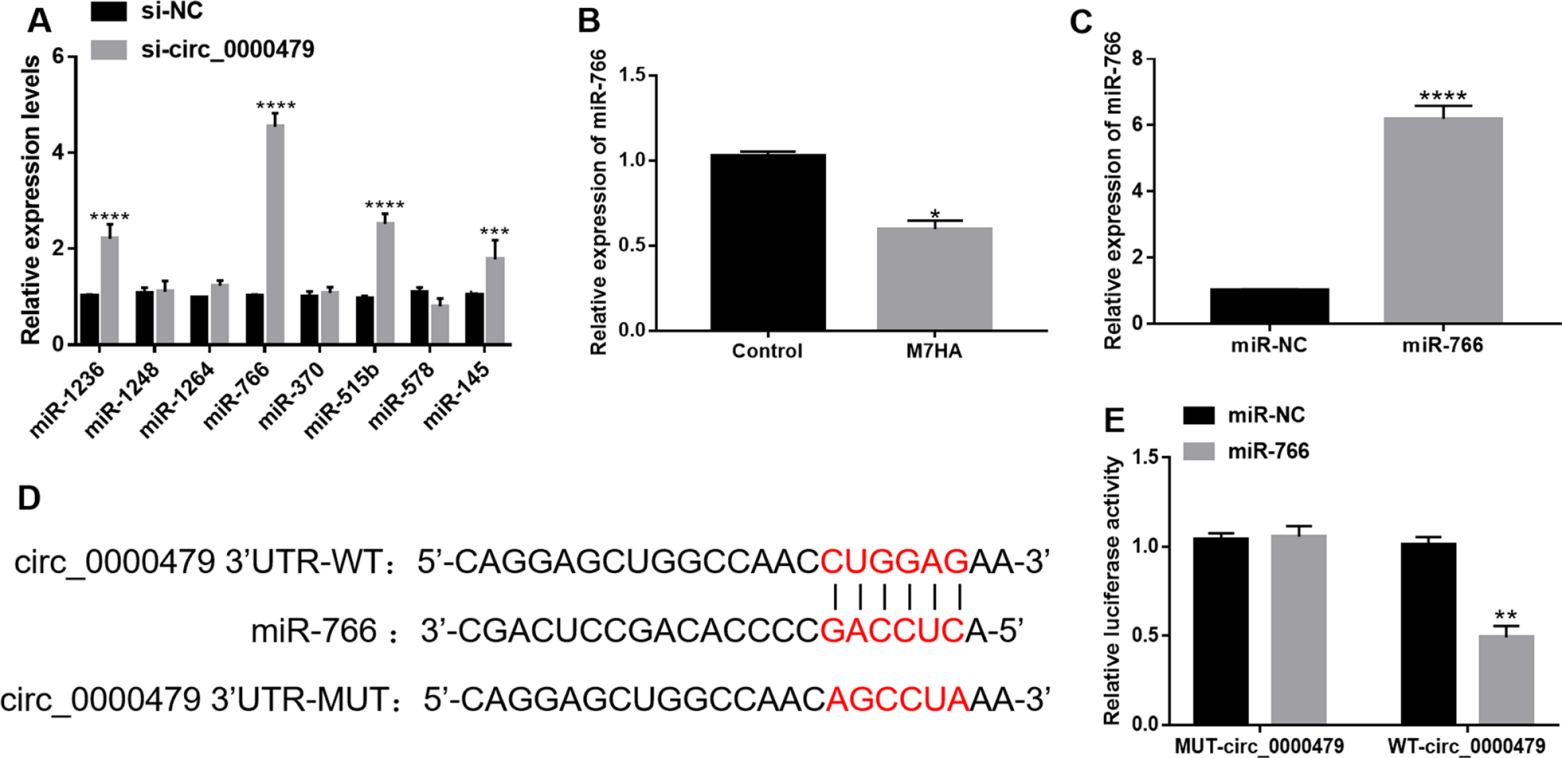
MiR-766 was the target of circ_0000479. **A** Detection of miRNA expression levels in MH7A cells by qRT-PCR. **B** Relative expression of miR-766 in MH7A cells and normal FLSs. **C** Relative expression of miR-766 in MH7A cells transfected with miR-NC or miR-766. **D** The binding sites between circ_0000479 and miR-766. **E** Relative luciferase activity of WT-circ_0000479 and MUT-circ_0000479 after transfection of miR-NC or miR-766. *P < 0.05; **P < 0.01; ***P < 0.001; ****P < 0.0001

Through bioinformatics prediction from the online website circinteractome (https://circinteractome.irp.nia.nih.gov/), we identified 18 miRNAs that could bind to circ_0000479. We selected eight of them and verified their expression in MH7A cells by qRT-PCR. Compared with the si-NC group, circ_0000479 knockdown most significantly up-regulated the expression of miR-766 which was selected for the follow-up study (Fig. 3A). As illustrated in Fig. 3B, the expression level of miR-766 in MH7A cells was lower than that in the normal group. Transfection of miR-766 mimics significantly increased miR-766 expression in MH7A cells (Fig. 3C). Figure 3D shows the binding sequence of circ_0000479 to miR-766. The results of dual-luciferase reporter assays demonstrated that miR-766 overexpression inhibited the luciferase activity of WT-circ_0000479 in MH7A cells, but not the luciferase activity of MUT-circ_0000479, indicating the combination between circ_0000479 and miR-766 (Fig. 3E). Collectively, circ_0000479 sponged miR-766 to downregulate the expression of miR-766 in MH7A cells.

### Circ_0000479 regulated MH7A cell progression by targeting miR-766

**Fig. 4:**
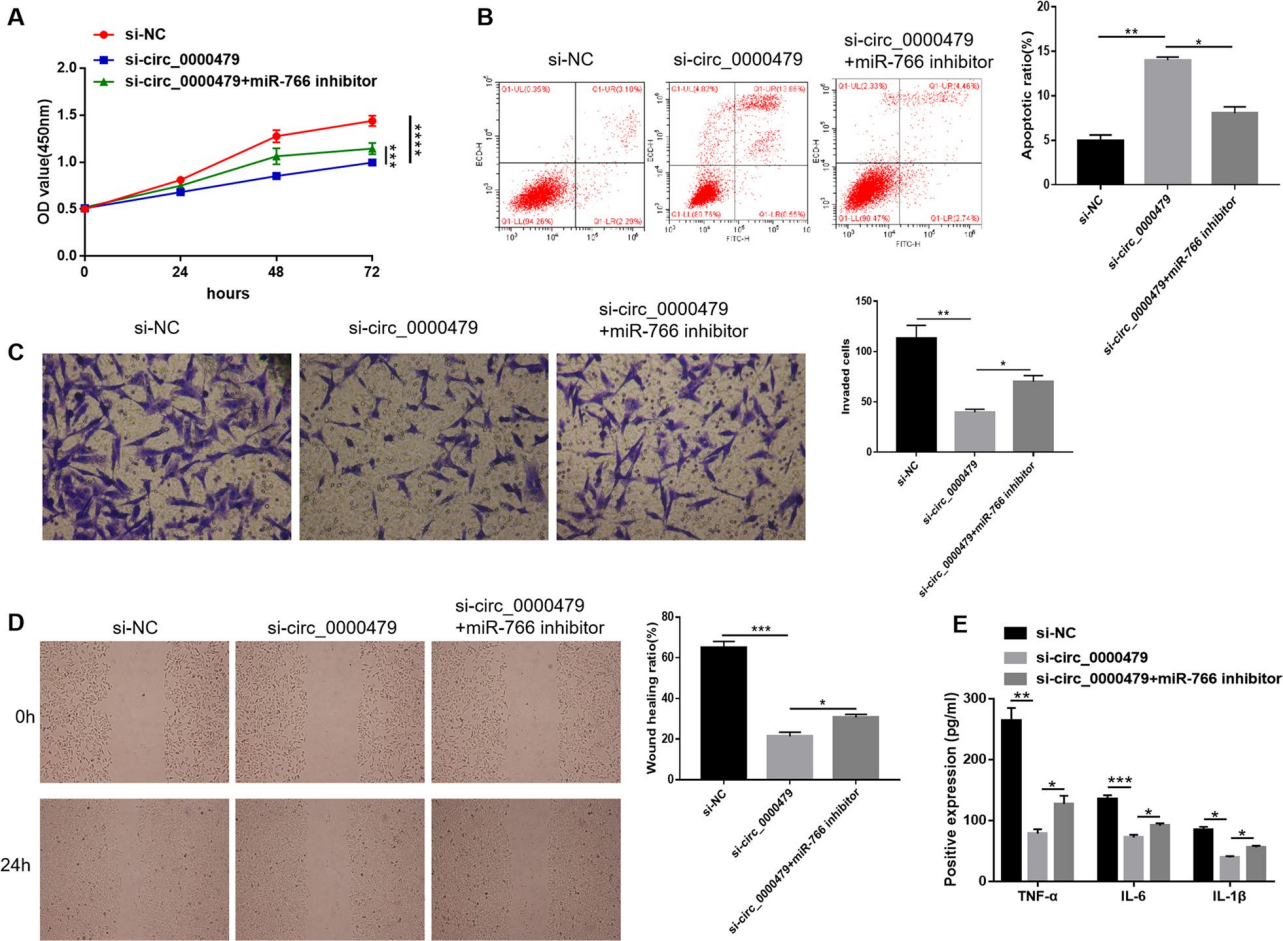
Circ_0000479 regulated MH7A cell progression by sponging miR-766. **A** The results of CCK-8 assays showed that the inhibitory effect of circ_0000479 knockdown on MH7A proliferation was abated by miR-766 inhibitor. **B** Flow cytometry analysis showed that circ_0000479 knockdown facilitated MH7A cell apoptosis, while miR-766 inhibitor attenuated the facilitation. **C**, **D** As demonstrated by transwell and wound-healing assays, circ_0000479 knockdown restrained the invasion and migration of MH7A cells, while miR-766 inhibition ameliorated the impacts. **E** circ_0000479 knockdown decreased the concentrations of TNF-α, IL-6 and IL-1β in MH7A cells, whereas miR-766 inhibition rescued the effects. Magnification 40 × for wound healing assays and 100 × for transwell assays. *P < 0.05; **P < 0.01; ***P < 0.001; ****P < 0.0001

To explore the roles of circ_0000479 and miR-766 in MH7A cell progression, we performed rescue experiments. As shown in Fig. 4A, the inhibitory effect of circ_0000479 knockdown on MH7A cell proliferation was attenuated by miR-766 inhibitor. Flow cytometry analysis showed that circ_0000479 knockdown promoted apoptosis in MH7A cells, whereas miR-766 inhibition abated the promotion (Fig. 4B). Circ_0000479 knockdown suppressed the invasion and migration of MH7A cells, whereas miR-766 inhibitor ameliorated the effects, as demonstrated by the results of transwell and wound healing assays (Fig. 4C, 4D). Moreover, miR-766 inhibition rescued the decreased concentrations of TNF-α, IL-6 and IL-1β in MH7A cells due to circ_0000479 knockdown (Fig. 4E). Taken all together, circ_0000479 knockdown suppressed MH7A cell progression by targeting miR-766.

### Target gene screening and validation of miR-766

**Fig. 5:**
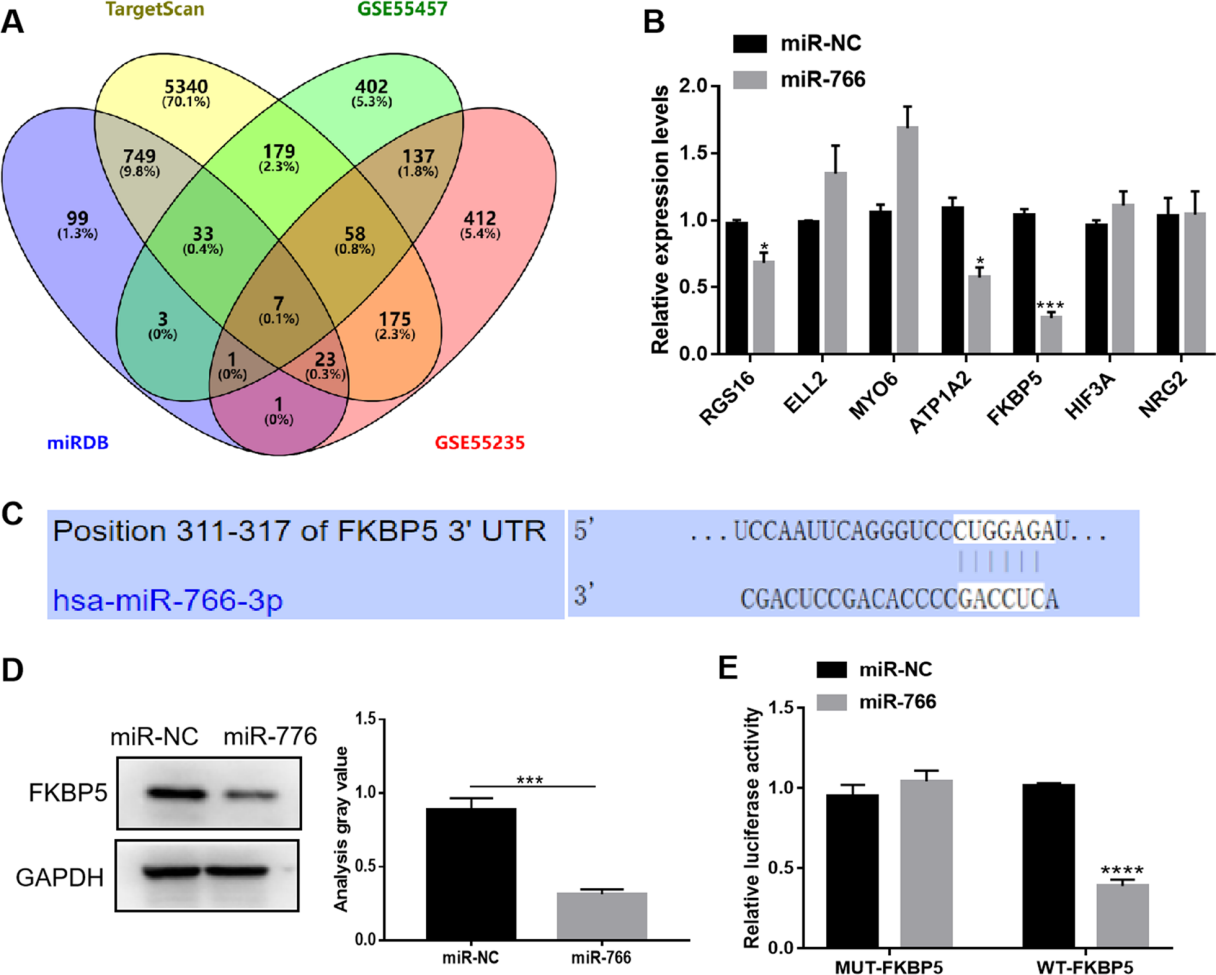
FKBP5 was the target of miR-766. **A** Venn diagram indicating the intersection of upregulated genes in RA and target genes of miR-766. **B** Relative expression levels of target genes of miR-766 by qRT-PCR. **C** The binding sites between miR-766 and FKBP5. **D** The expression level of FKBP5 protein was significantly down-regulated after miR-766 overexpression. **E** Relative luciferase activity of WT-FKBP5 and MUT-FKBP5 after transfection of miR-NC or miR-766. *P < 0.05; ***P < 0.001; ****P < 0.0001

We obtained the target genes of miR-766 by analysis in the miRDB and TargetScan databases. Meanwhile, we found datasets GSE55457 and GSE55235 from the GEO database, and screened out the genes that were significantly up-regulated in RA. These up-regulated genes were then intersected with the target genes of miR-766 using Venn diagram Venny2.1, resulting in a total of 7 genes (Fig. 5A). Next, we screened the target genes by qRT‑PCR, and the results showed that FKBP5 was most significantly down-regulated after miR-766 overexpression (Fig. 5B). The binding sequence of miR-766 and FKBP5 predicted by TargetScan database is shown in Fig. 5C. It was further verified at the protein level by western blot analysis. After overexpression of miR-766, the protein expression level of FKBP5 was significantly down-regulated, as shown in the grayscale analysis in Fig. 5D. The results of dual-luciferase reporter assays showed that miR-766 overexpression inhibited the luciferase activity of WT-FKBP5 in MH7A cells, but not the luciferase activity of MUT-FKBP5, indicating that miR-766 could target the 3’UTR of FKBP5 (Fig. 5E).

### Circ_0000479 affected MH7A cell progression through FKBP5

**Fig. 6:**
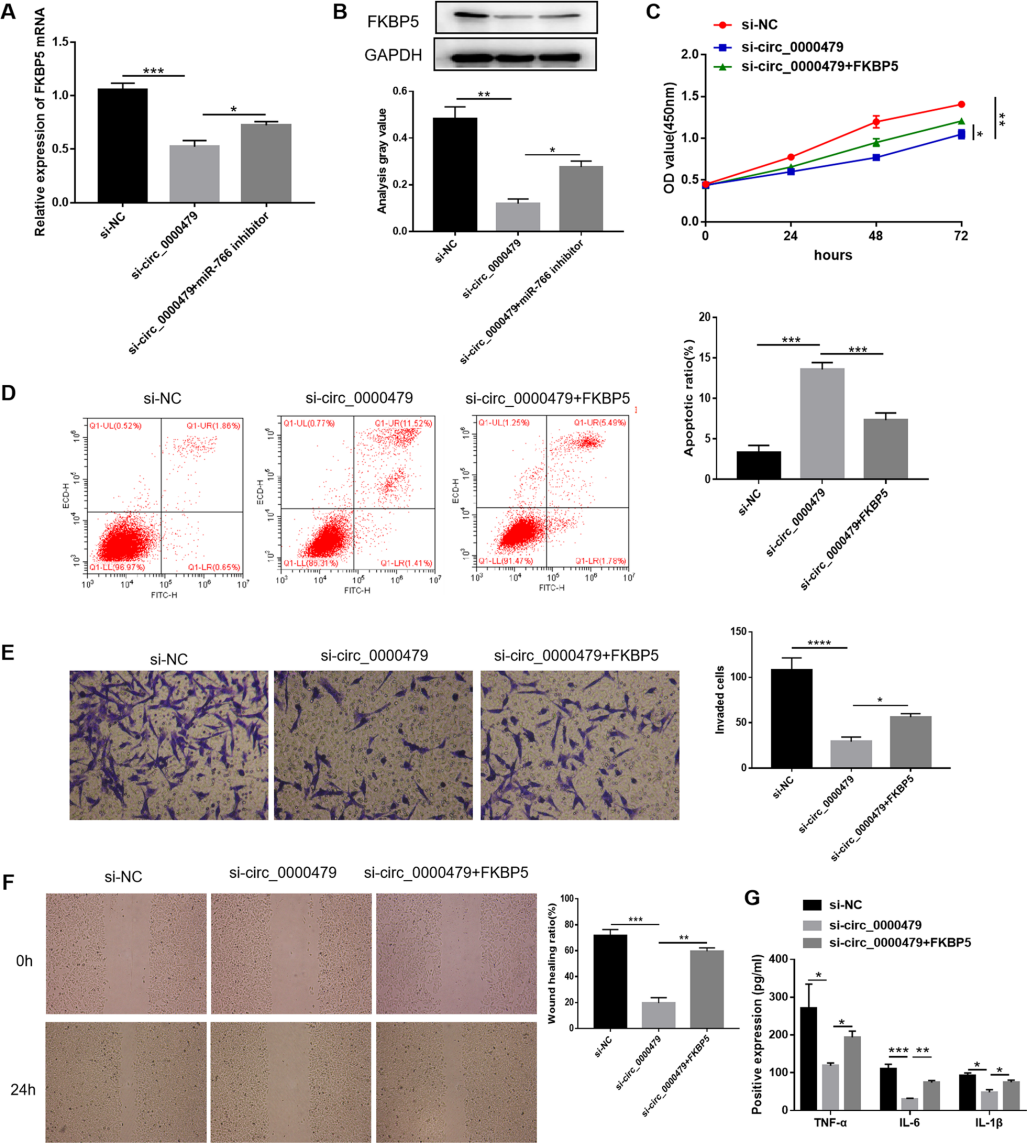
Circ_0000479 affected MH7A cell progression through FKBP5. **A** **B** The relative mRNA and protein levels of FKBP5 in MH7A cells were measured by qRT-PCR and western blot analysis. **C**, **D** The proliferation and apoptosis of MH7A cells were estimated by CCK-8 and flow cytometry analysis. **E**, **F** The invasion and migration of MH7A cells were assessed by transwell and wound-healing assays. **G** The positive expression of TNF-α, IL-6 and IL-1β in MH7A cells were examined by ELISA kits. Magnification 40 × for wound healing assays and 100 × for transwell assays. *P < 0.05; **P < 0.01; ***P < 0.001; ****P < 0.0001

Finally, we further analyzed the relationship among circ_0000479, miR-766 and FKBP5. Silencing of circ_0000479 reduced the mRNA and protein expression levels of FKBP5, while the expression of FKBP5 increased to a certain extent after adding miR-766 inhibitor, indicating that circ_0000479 regulated the expression of FKBP5 through miR-766 (Fig. 6A, B). Knockdown of circ_0000479 inhibited the proliferation, invasion and migration of MH7A cells, and its effect was abated after co-transfection with FKBP5 overexpression vector (Fig. 6C, E, F). Flow cytometry analysis demonstrated that circ_0000479 silencing accelerated MH7A cell apoptosis, which was attenuated by upregulation of FKBP5 (Fig. 6D). Circ_0000479 knockdown decreased the concentrations of TNF-α, IL-6 and IL-1β in MH7A cells, while FKBP5 overexpression alleviated this impact (Fig. 6G). Therefore, we conclude that circ_0000479 knockdown suppressed MH7A cell progression through FKBP5.

## Discussion

First discovered more than 40 years ago, circRNAs were once thought to be errors in the normal splicing process. With advances in high-throughput RNA sequencing and circRNA-specific bioinformatics algorithms, circRNAs have been found to be important players in normal cell differentiation and tissue homeostasis, as well as in disease development [12, 23]. By interacting with disease-related miRNAs, circRNAs play an important regulatory role in diseases, which is the main research idea of circRNAs at present [24–26]. In this study, we found that circ_0000479 was upregulated in RA-FLSs while its parental gene EPSTI1 was not differentially expressed, and circ_0000479 knockdown inhibited proliferation, invasion, migration and infammation and promoted apoptosis of RA-FLSs. Guo et al. found that circ_0000479 regulates SLE progression by modulating metabolic pathways and the Wnt signaling pathway [20]. In the following research, we further explored the specific mechanism of action of circ_0000479 in RA progression.

Accumulating studies have shown that the circRNA/miRNA/mRNA axis is closely related to the occurrence and development of RA [19, 27, 28]. Through bioinformatics analysis and screening, we obtained 18 miRNAs, of which miR-766 was most significantly up-regulated after circ_0000479 knockdown. Meanwhile, miR-766 was also confirmed to be down-regulated in RA-FLSs and the combination between circ_0000479 and miR-766 was verified by dual‑luciferase reporter assays. Cai et al. presented that circ_0088194 promotes the invasion and migration of RA-FLSs via the miR-766-3p/MMP2 axis [27]. And miR-766-3p contributes to anti-inflammatory responses in RA-FLSs by indirectly reducing the activation of NF-κB [29]. By performing rescue experiments, we illustrated that circ_0000479 knockdown suppressed the progression and inflammation of RA-FLSs by regulating miR-766.

Subsequently, we conducted target gene screening and validation of miR-766. By intersecting the target genes of miR-766 with the up-regulated genes in RA, we obtained a total of 8 genes, of which FKBP5 was most significantly down-regulated after miR-766 overexpression. In addition, the results of dual-luciferase reporter assays verified that miR-766 could target the 3’UTR of FKBP5. FKBP5 is a member of the immunophilin protein family, which play a role in immunoregulation and basic cellular processes involving protein folding and trafficking [30]. Recent studies have disclosed that FKBP5 is upregulated in bone marrow mononuclear cells from RA patients [31, 32], and FKBP5 has a direct positive effect on osteoclast differentiation and may play a role in the development of bone destruction and osteoporosis in RA [33]. In this study, we demonstrated that silencing of circ_0000479 suppressed the RA-FLS progression through FKBP5 via rescue experiments. However, the miR-766 inhibitor or FKBP5 overexpression could not completely reverse the effect of circ_0000479 knockdown on MH7A cell function, suggesting that other mechanisms may exist. Since si-circ_0000479 was proved not to affect the expression of the parental gene EPSTI1, we speculated that circ_0000479 may not affect the pathogenesis of RA by regulating the expression of EPSTI1 in this study, but this does not negate the potential function of EPSTI1 in the pathogenesis of RA.

In summary, this study revealed a novel mechanism whereby circ_0000479 directly binds to miR-766 to promote proliferation, invasion, migration and inflammation and inhibit apoptosis of RA-FLSs via FKBP5. This work contributes to a better understanding of the role of circ_0000479 in RA, and suggests that reducing the level of circ_0000479 may be a novel approach for the treatment of RA. Despite these promising results, limitations remain. Only in vitro experiments were performed in cell lines, and no further validation was conducted in samples from RA patients and animal models. In addition, whether circ_0000479 can affect RA progression through other pathways has not been studied, which is also the direction of future research.

## Data Availability

Data supporting the findings of this study are available from the corresponding author upon reasonable request.
